# Informed Consent for Pediatric Lyme Meningitis Study: In‐Person Versus Remote Approach

**DOI:** 10.1111/acem.70400

**Published:** 2026-09-03

**Authors:** Christian D. Pulcini, Thomas H. Chun, Jonathan A. Race, Desiree N. Neville, Rebecca S. Green, Andrew S. Handel, Courtney E. Coyle, Paul L. Aronson, Amy D. Thompson, Sunil K. Sood, Alexandra B. Yonts, Kathryn E. Kasmire, Christoper Woll, Anna R. Huppler, Christina R. Hermos, Christina Gagliardo, Mariann N. Kelley, Anupam B. Kharbanda, Sheila M. Nolan, Margaret E. Samuels‐Kalow, Maia S. Rutman, Lise E. Nigrovic, Pedi Lyme Net Lyme Meningitis Study Group

**Affiliations:** ^1^ Department of Emergency Medicine and Pediatrics University of Vermont Medical Center Burlington Vermont USA; ^2^ Department of Emergency Medicine and Pediatrics Rhode Island Hospital Providence Rhode Island USA; ^3^ Department of Pediatrics Utah School of Medicine Salt Lake City Utah USA; ^4^ Division of Emergency Medicine Children's Hospital of Pittsburgh of UPMC Pittsburgh Pennsylvania USA; ^5^ Division of Emergency Medicine Children's Hospital of Philadelphia Philadelphia Pennsylvania USA; ^6^ Division of Infectious Diseases Stony Brook University Medical Center Stony Brook New York USA; ^7^ Division of Emergency Medicine Nationwide Children's Hospital Columbus Ohio USA; ^8^ Section of Pediatric Emergency Medicine Yale New Haven Medical Center New Haven Connecticut USA; ^9^ Division of Emergency Medicine Nemours Children's Hospital Wilmington Delaware USA; ^10^ Division of Infectious Diseases Northwell Cohen Children's New Hyde Park New York USA; ^11^ Division of Infectious Diseases Children's National Hospital Washington District of Columbia USA; ^12^ Departments of Emergency Medicine and Pediatrics Penn State Health Hershey Medical Center Hershey Pennsylvania USA; ^13^ Departments of Emergency Medicine and Pediatrics Albany Medical Center Albany New York USA; ^14^ Department of Pediatrics Children's Wisconsin Milwaukee Wisconsin USA; ^15^ Division of Infectious Diseases UMass Memorial Children's Medical Center Worcester Massachusetts USA; ^16^ Division of Pediatric Infectious Diseases Goryeb Children's Hospital, Atlantic Health System Morristown New Jersey USA; ^17^ Division of Pediatric Emergency Medicine University of Connecticut School of Medicine, Connecticut Children's Hartford Connecticut USA; ^18^ Department of Pediatric Emergency Medicine Children's Minnesota Minneapolis Minnesota USA; ^19^ Division of Pediatric Infectious Diseases Westchester Medical Center Valhalla New York USA; ^20^ Department of Emergency Medicine Mass General Brigham Boston Massachusetts USA; ^21^ Department of Pediatrics and Emergency Medicine Dartmouth‐Hitchcock Medical Center Lebanon New Hampshire USA; ^22^ Division of Emergency Medicine Boston Children's Hospital Boston Massachusetts USA

Traditional in‐person enrollment is not always feasible for clinical research, notably when eligibility confirmation is delayed [1]. Remote enrollment, including telephone‐based approaches, offer a potential solution to this challenge while maintaining the ethical principles of informed consent. This approach is understudied in children despite known challenges in recruiting pediatric subjects in clinical research studies [2].

To address this knowledge gap, we evaluated remote versus in‐person approaches on enrollment rates using a recently completed 21‐center prospective observational study comparing intravenous ceftriaxone to oral doxycycline on time to symptom resolution for children with Lyme meningitis (details described previously) [3]. The single Institutional Review Board (sIRB) at Boston Children's Hospital designated the study as minimal risk and established reliance agreements with each participating center.

We included children aged 1 to 21 years with meningitis and positive two‐tier Lyme disease serology with a provider plan to treat with either intravenous ceftriaxone or oral doxycycline. We did not include children whose Lyme disease serology or treatment initiation was more than 7 days ago or who could not complete study surveys. Because Lyme disease serology typically required several days to return results, potentially eligible patients received a study information sheet from either the study or clinical team and were asked for verbal permission for later contact. Those who did not provide consent for later contact were considered ineligible.

Once eligibility was confirmed, study coordinators approached caregivers (for children under 18 years) or patients (ages 18–21 years) either in‐person during a clinical encounter or remotely by telephone. Consent documents and surveys were available in either English or Spanish. For children older than 7 but under 18 years of age, assent was obtained in accordance with institutional policies. Antibiotic treatment was selected by treating clinicians with study follow‐up by electronic surveys, telephone interview, and medical record review. Study staff followed a standard consent script for both in‐person and remote encounters and allowed children and their caregivers the opportunity to ask questions before providing informed consent.

First, we used Spearman's correlation coefficient with 95% confidence intervals (CIs) to measure the relationship between the number of parents/patients approached and enrollment rates by participating site. Second, we compared enrollment rates between remote and in‐person consent approaches using descriptive statistics and 95% CIs for the difference in proportions. We used SAS version 9.4 for all analyses.

Of 360 eligible patients, study teams approached 302 (83.9%) for consent (Figure 1). Documented reasons for non‐approach included inability to contact parents (*n* = 38), unavailable research staff (*n* = 2), and clinician request (*n* = 1). The number approached varied by participating center (range 2–72 children). Among those approached, 253 participants consented (83.8% consent rate), which was inversely correlated with number of parents/patients approached (−0.51 Spearman's coefficient, 95% CI −0.79 to −0.11; *p* = 0.02).

**FIGURE 1:**
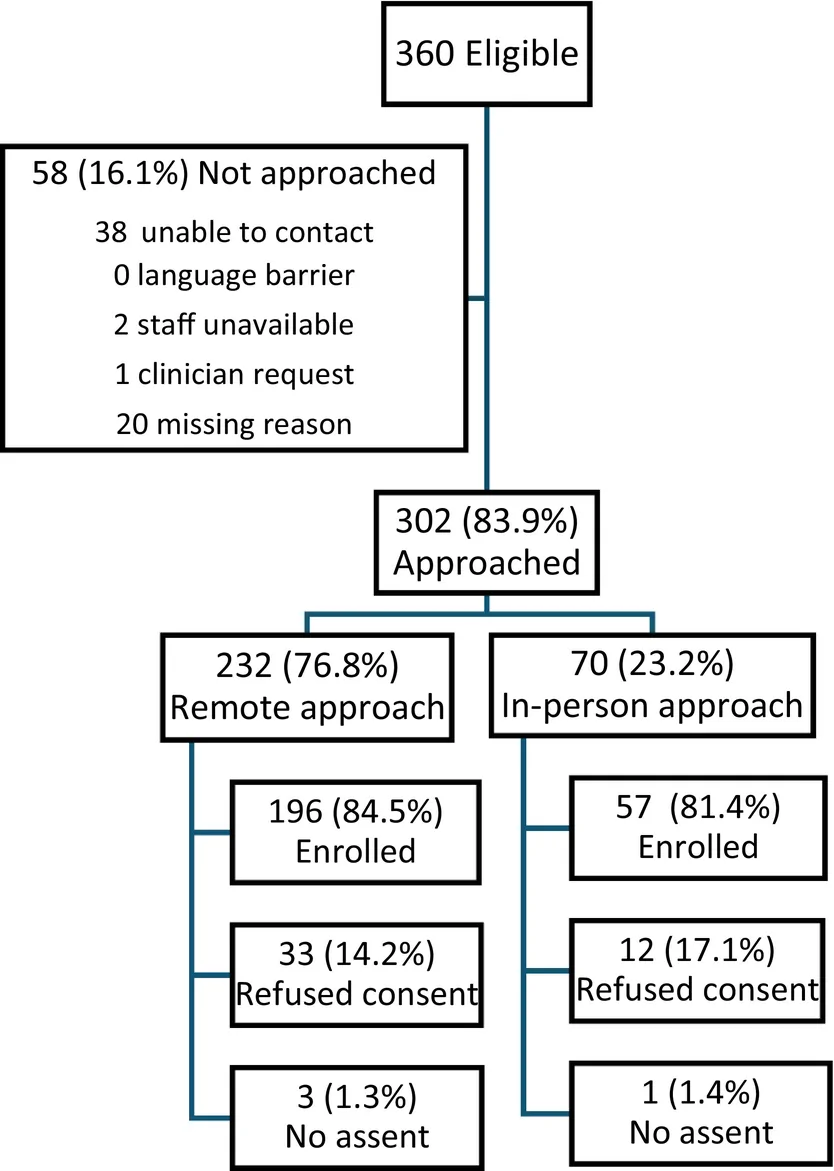
Study patients for Lyme Meningitis Study: In‐person versus remote approach.

Of the 302 parents/patients approached for enrollment, 232 (76.8%) were approached remotely and 70 (23.2%) were approached in‐person (Figure 1). Four children did not provide assent and were not enrolled (3 remote and 1 in‐person approach). Enrollment rates were similar between remote and in‐person approaches: 84.4% (196/232) remote versus 81.4% (57/70) in‐person (difference 3.1%, 95% CI −6.0% to 14.5%). Three enrolled children who were later found ineligible were withdrawn (1 remote and 2 in‐person consents).

In our recently completed multi‐center observational study of children with Lyme meningitis, the remote telephone‐based approach had comparable consent rates to in‐person approach rates. The emergency department served as the primary site for initial patient identification: children presenting with suspected Lyme meningitis were flagged by the clinical or study team during their initial encounter, and study information sheets were provided at that time before eligibility could be confirmed. This front‐end screening ensured that no eligible patients were missed during the window between presentation and laboratory confirmation. Given that most children had study eligibility confirmation after the initial clinical encounter, the remote approach proved essential for completing enrollment. The high approach and consent rates (above 80%) demonstrate the effectiveness of this model and may be broadly applicable to other studies using verbal or electronic consent platforms. The number of children approached varied considerably between centers, reflecting local Lyme disease incidence as well as study team availability. Importantly, consent rates were higher for centers with a low number of patients approached, demonstrating the ability of all centers to make meaningful contributions to clinical studies of uncommon conditions such as Lyme meningitis.

Several factors may explain the comparable success of remote consent in this study. First, families had already received preliminary information about the study during the initial clinical encounter, allowing time to consider study participation before the formal consent discussion. Additionally, patients and families granted permission for later contact at the time of the in‐person encounter if later found eligible. This staged, clinically embedded approach of introducing the study during the initial encounter to secure permission for later contact connects research recruitment directly to clinical care. Compared to post‐discharge contact alone, this approach benefits families by offering multiple opportunities to ask questions and time to consider participation while streamlining recruitment for research staff. Second, keeping treatment decisions with the clinical care team as opposed to dictated by the study protocol may have increased willingness to consider participation. Third, the sIRB waiver of written documentation of informed consent for remote approaches reduced the logistical challenges associated with telephone consent. Finally, intense media coverage of Lyme disease risks, including persistent symptoms despite appropriate treatment, may have increased patient and parental interest in participation [4, 5].

Our findings align with previous research demonstrating the feasibility and acceptability of remote consent approaches [6]. Telemedicine consent can achieve non‐inferior comprehension compared to face‐to‐face consent in emergency department settings [7]. During the COVID‐19 pandemic, electronic and remote consent methods became essential for continuing clinical studies, while minimizing in‐person contact [8]. Our study extends this evidence to telephone‐based consent for pediatric populations, notably when eligibility confirmation is delayed.

Important considerations remain for implementing remote consent in clinical studies enrolling children. Ensuring equitable access requires accommodating families without consistent telephone access or those preferring in‐person discussions [9]. Language barriers may pose additional challenges for remote consent, potentially requiring interpreter services via three‐way calling [10]. While remote consent methods are non‐inferior to in‐person methods [7], future research should evaluate optimal timing for post‐encounter contact, preferred communication methods among diverse populations, and study characteristics that make remote or in‐person consent more appropriate [2].

In addition to lessons learned on remote versus in‐person enrollment, our study also highlights the importance of establishment and maintenance of large, multi‐center collaborations when studying uncommon conditions or rare outcomes such as Lyme meningitis in children [11]. Individual centers rarely encounter sufficient cases of rare diseases to complete adequately powered studies within a reasonable timeframe. Multi‐center networks therefore play a critical role in aggregating patients across geographic regions and practice settings. In our study, increasing the diversity of participating centers, including urban and rural sites as well as both freestanding children's hospitals and general hospitals where children receive care, enabled enrollment of patients from a broad range of Lyme‐endemic regions in the United States. Importantly, although the number of participants approached varied across sites, enrolled patients came from a wide range of participating centers. Our findings underscore that even centers with relatively small case volumes can meaningfully contribute to multi‐center studies of uncommon pediatric conditions. Building and sustaining broad research networks is therefore essential to generating broadly applicable evidence for diseases such as Lyme meningitis, where single‐center studies would be unlikely to achieve sufficient sample size.

Our study must be interpreted in the context of its limitations. First, we were unable to determine the impact of the location of approach on child assent rates as only a minority of children failed to provide assent. Second, we did not capture site and study staff characteristics which may have impacted enrollment rates. Last, we were underpowered to detect small differences in consent rates. Additionally, the waiver of documentation of written informed consent was permitted given the minimal‐risk designation. Therefore, our findings may not be applicable to greater than minimal risk studies which require written documentation of consent. For such studies, electronic consent platforms that deliver written and potentially video‐enhanced information to support a remote consent discussion may offer a viable alternative to fully in‐person consent.

In the Lyme meningitis study, our high approach and enrollment rate (both above 80%) suggest that advance notification combined with flexible consent approaches can effectively support recruitment of children in a clinical study. When confirmation of study eligibility can occur after the initial encounter, remote consent provides an effective tool for maximizing research participation by capturing all eligible patients. In our study, remote and in‐person enrollment rates did not differ, suggesting the location of approach should be determined by logistical considerations and family preferences rather than concerns about enrollment success. Future studies should evaluate optimal methods for post‐encounter contact with families and determine which study characteristics are best suited for remote versus in‐person consent approaches.

## Author Contributions

L.E.N. and T.H.C. conceived and designed the project. J.A.R. performed data analysis. C.D.P., T.H.C., D.N.N., R.S.G., A.S.H., C.E.C., P.L.A., A.D.T., S.K.S., A.B.Y., K.E.K., C.W., A.R.H., C.R.H., C.G., M.N.K., A.B.K., S.M.N., M.E.S.‐K., and M.S.R. participated in participant recruitment and data collection. C.D.P. and L.E.N. drafted the manuscript and coordinated edits. All authors provided critical input during development of the manuscript and approved the final version. The authors are responsible for the correctness of the statements provided in the manuscript.

## Funding

This research was supported by the National Institute of Allergy and Infectious Diseases (NIAID R01A151180‐02A1).

## Data Availability

The data that support the findings of this study are available from the corresponding author upon reasonable request.
